# Evaluation of a low-cost, portable imaging system for early detection of oral cancer

**DOI:** 10.1186/1758-3284-2-10

**Published:** 2010-04-22

**Authors:** Mohammed S Rahman, Nilesh Ingole, Darren Roblyer, Vanda Stepanek, Rebecca Richards-Kortum, Ann Gillenwater, Surendra Shastri, Pankaj Chaturvedi

**Affiliations:** 1 Department of Bioengineering, Rice University, Houston, 77005, USA; 2 Head and Neck Surgery, Tata Memorial Hospital, Mumbai, 400012, India; 3 Department of Head and Neck Surgery, University of Texas M.D. Anderson Cancer Center, Houston, 77030, USA

## Abstract

**Background:**

There is an important global need to improve early detection of oral cancer. Recent reports suggest that optical imaging technologies can aid in the identification of neoplastic lesions in the oral cavity; however, there is little data evaluating the use of optical imaging modalities in resource limited settings where oral cancer impacts patients disproportionately. In this article, we evaluate a simple, low-cost optical imaging system that is designed for early detection of oral cancer in resource limited settings. We report results of a clinical study conducted at Tata Memorial Hospital (TMH) in Mumbai, India using this system as a tool to improve detection of oral cancer and its precursors.

**Methods:**

Reflectance images with white light illumination and fluorescence images with 455 nm excitation were obtained from 261 sites in the oral cavity from 76 patients and 90 sites in the oral cavity from 33 normal volunteers. Quantitative image features were used to develop classification algorithms to identify neoplastic tissue, using clinical diagnosis of expert observers as the gold standard.

**Results:**

Using the ratio of red to green autofluorescence, the algorithm identified tissues judged clinically to be cancer or clinically suspicious for neoplasia with a sensitivity of 90% and a specificity of 87%.

**Conclusions:**

Results suggest that the performance of this simple, objective low-cost system has potential to improve oral screening efforts, especially in low-resource settings.

## Background

Oral cancer is a major health problem worldwide, causing over 127,000 deaths each year [1]. With an annual incidence exceeding 274,000 cases, oral cancer ranks as one of the top ten most common malignancies. Over two-thirds of cases and three-quarters of deaths due to oral cancer occur in developing countries [2]. In the U.S., the overall five-year-survival rate for patients with oral cancer is only 54%, one of the lowest rates of all major cancers; in developing countries, five-year survival rates drop below 30% [1,3]. Patients with early lesions have better chances for cure and less treatment associated morbidity, yet despite the easy accessibility of the mouth, most patients present with advanced tumors, when treatment is more difficult, more expensive and less successful compared to earlier interventions. Early detection of oral premalignant lesions (OPLs) and early neoplastic changes may be our best and most cost-effective means to improve survival and quality of life for oral cancer patients from all socioeconomic communities [3].

No satisfactory mechanism exists currently to screen and detect early neoplastic changes of the oral cavity in the general population. Possible explanations include: 1) There is limited public awareness and insufficient education of health care workers about oral cancer risk factors, signs and symptoms. 2) Detection relies heavily on clinical experience at recognition of suspicious lesions during physical examination, with variable effectiveness [4,5]. It can be difficult to distinguish OPLs from more common inflammatory conditions. 3) Practitioners and patients are reluctant to perform invasive biopsies of oral lesions which are expensive and often require referral to a specialist. 4) Some patients have field cancerization, where the entire mucosal lining that is exposed to carcinogens in alcohol and tobacco sustains damage and is at risk to develop cancer [6]. In high risk patients, often the whole lining of the oral cavity is potentially premalignant, making it difficult even for experienced clinicians to know when and where to biopsy [5]. The situation is even more challenging in developing countries and low-resource regions with high-risk populations, where a combination of lack of public awareness of the disease and inadequate resources and expertise for screening can result in even greater delays in diagnosis, leading to higher morbidity and mortality. One recent study conducted in southern India concluded that even simple visual examination by health workers can prevent 37,000 oral cancer deaths annually worldwide [7].

Recent advances in optical imaging have the potential to improve early detection of oral cancer and its precursors. Several groups have demonstrated that imaging systems that record the spatial distribution of tissue fluorescence at specific excitation/emission wavelength combinations can be used to survey large areas of oral cavity mucosa to non-invasively detect early changes associated with oral cancer in real-time [5,7-12]. Lane presented a non-magnifying hand-held device for direct visualization of oral cavity tissue fluorescence, which is now FDA approved for clinical use and commercially available as the VELscope^® ^[5]. The system uses a metal-halide lamp with emission peaks at 405 and 436 nm to excite autofluorescence; images viewed by eye through the VELscope indicate a characteristic loss of fluorescence associated with malignant progression. Results from 50 biopsies taken from areas with loss of fluorescence in 44 patients showed a sensitivity of 98% and specificity of 100% for discriminating normal tissue from severe dysplasia, carcinoma *in *situ, or invasive carcinoma, using histology as the gold standard. An important finding was the ability of fluorescence visualization to aid clinicians in identifying early neoplastic lesions that were initially missed during traditional white light examination (WLE) [11].

To address the need for objective image interpretation, Roblyer recently described a multimodal digital microscope to obtain digital images of oral tissue fluorescence and reflectance and evaluated the ability of objective image analysis techniques to recognize OPLs and early oral cancer. Using 405 nm excited fluorescence images, algorithms could detect OPLs and oral cancer with a sensitivity of 94% and specificity of 87% in 64 patients [12]. The results of automated analysis of digital autofluorescence images must also be validated in larger clinical trials.

While digital image analysis can provide more objective interpretation, lower cost devices are needed for use in developing countries. To address the need for low-cost imaging, Rahman recently developed a multi-modal imaging system for screening and detection of oral cancer in high-risk populations in low-resource and remote settings [13]. This portable, battery powered system acquires near real-time digital images of oral tissue in reflectance and fluorescence mode. In this paper, we report results of a clinical study to evaluate the ability of this device to aid in detection of oral cancer in India involving 109 subjects at high-risk for developing oral cancer. We also characterize the optical properties of two types of potentially confounding oral lesions, melanosis and oral submucous fibrosis (OSF), which are common in this region, and discuss how the presence of these lesions influences the ability of the device to accurately identify neoplastic lesions.

## Methods

### Instrumentation

The portable imaging system used in this study consisted of a modified commercial headlamp system; details of the device have been described previously [13]. Briefly, the multi-modal imaging system uses light emitting diodes (LEDs) to illuminate the oral mucosa. For fluorescence imaging, the system has a blue LED with an excitation peak at 455 nm wavelength; for reflectance imaging, it has a white LED with an illumination range of 400 to 700 nm. Images can either be observed visually or captured digitally through a set of optical filters using an integrated, miniature charge coupled device (CCD) camera. The system is connected to a laptop via a firewire interface to record and store the images. The portable system weighs only 3 pounds and can be powered by a lithium-ion battery.

### Protocol and Image Acquisition

The study was conducted at Tata Memorial Hospital (TMH) in Mumbai, India. Patients who were referred to the Cancer Prevention Clinic at Tata Memorial Hospital because of suspicious oral lesions or were waiting for head & neck surgery in the hospital ward were recruited to participate in the study. In addition to patients, healthy volunteers with and without a history of using tobacco were recruited to participate in the study. The clinical study was reviewed and approved by the Hospital Ethics Committee (HEC) at TMH and the image analysis study was reviewed and approved by the Institutional Review Board at Rice University. Written informed consent was obtained from each subject enrolled in the study.

*In vivo *imaging measurements from subjects were obtained in the Cancer Prevention Clinic. All measurements were taken in a darkened room to avoid room light interference. The imaging system was positioned approximately 20 cm away from the subjects. Reflectance image exposure was a few milliseconds while fluorescence image exposure was approximately 500 milliseconds.

A head & neck specialist at the clinic assessed each participating patient by conducting a conventional examination of the oral cavity. Initial clinical impression of each site as normal or abnormal was noted. In addition, the presence of either melanosis - darkly pigmented lesions - or oral submucous fibrosis - rigid, fibrotic white lesions - was noted if visible. After clinical examination, digital reflectance and fluorescence images were obtained from clinically abnormal sites and contralateral clinically normal sites. Images were also obtained from the lateral border of the tongue, the buccal mucosa, and the lip of each subject whenever these sites were accessible. A quality control check was performed on all images before further analysis. Sites with poor image quality (e.g. out-of-focus images) were excluded from analysis.

For sites with an initial clinical impression of abnormal, the white light reflectance images were reviewed by three expert observers who were blinded to the fluorescence images (NI, AG, PC). At each site, the observer assigned a single clinical impression of 'Normal', 'Low Risk for Neoplasia', 'High Risk for Neoplasia', or 'Cancer'. Consensus clinical impression was used to determine the final diagnostic category for each site imaged. In cases where the impression of one of the expert observers differed from the other two, the clinical impression assigned by two of the three observers was used as the consensus. Measurements in which all three observers disagreed on the clinical impression were excluded from the analysis; a total of four sites were excluded for this reason. Sites with an initial clinical impression of normal were categorized with a diagnosis of 'Normal'. Performances of algorithms based on features of digital optical image analysis are reported relative to this consensus clinical impression.

### Image Analysis

White light reflectance images of each site were first examined; if the area was clinically abnormal, a region of interest (ROI) corresponding to the lesion was defined. If the area was clinically normal, a representative ROI was selected from the white light reflectance image. The same ROI was identified in each color fluorescence image of that site, and quantitative image features were calculated for each ROI.

Reflectance and fluorescence images were analyzed to yield possible features for use in classification algorithms. The following metrics were generated for ROIs corresponding to lesions and contralateral normal measurements: the average intensity in the red, green and blue (RGB) channels, average values of the ratios of the R/G, R/B and B/G intensities, the average intensity following grayscale conversion, and the standard deviation of the RGB and grayscale intensity values. In addition, for each ROI corresponding to a clinically abnormal site, we calculated the ratio of the metric for the lesion relative to that measured from the contralateral normal ROI in the same patient. We refer to these metrics as 'normalized ratios'. For measurements from clinically normal sites, normalized ratios were obtained by dividing each ROI in two and calculating the ratio of the metrics from the two resulting regions.

We explored which of these features provided the best separation between non-neoplastic oral mucosa and neoplastic oral mucosa. For calculation of sensitivity and specificity, sites with a diagnosis of 'Cancer' or 'High Risk' were considered to be neoplastic, while sites with a clinical diagnosis of 'Normal' or 'Low Risk' were considered to be non-neoplastic. Prior to feature selection, sites with a clinical descriptor of melanosis were excluded from the data set. Binary classification algorithms were developed using linear discriminant analysis with a single image feature as input. The same clinical dataset was used to both develop the algorithm and to assess classification accuracy. For each image feature, diagnostic performance was assessed as the threshold was varied from the minimum to the maximum of its value to generate a receiver operating characteristic (ROC) curve. Classification performance measures, such as the area under the receiver operating curve (AUC), sensitivity and specificity at the Q-point, were calculated for each of the input metrics using consensus clinical impression as the gold standard.

## Results

Images from a total of 351 different sites were included in the analysis (Table 1). 261 of these sites were imaged from 76 patients and 90 sites were imaged from 33 normal volunteers. A total of 222 sites had a consensus clinical impression of normal, 30 sites had a consensus clinical impression of low risk for neoplasia, 22 sites had a consensus clinical impression of high risk for neoplasia, and 37 had a consensus clinical impression of cancer. Melanosis was noted in 30 sites, and oral submucous fibrosis was visible in ten sites.

**Table 1:** Consensus Clinical Impression of Measured Sites

	Consensus Clinical Impression			
	**Normal**	**Low Risk for Neoplasia**	**High Risk for Neoplasia**	**Cancer**

**Melanosis or OSF Not Visible**	222	30	22	37
**Visible Melanosis**	8	22	0	0
**Visible OSF**	2	8	0	0
**Total**	232	60	22	37

**Figure 1:**
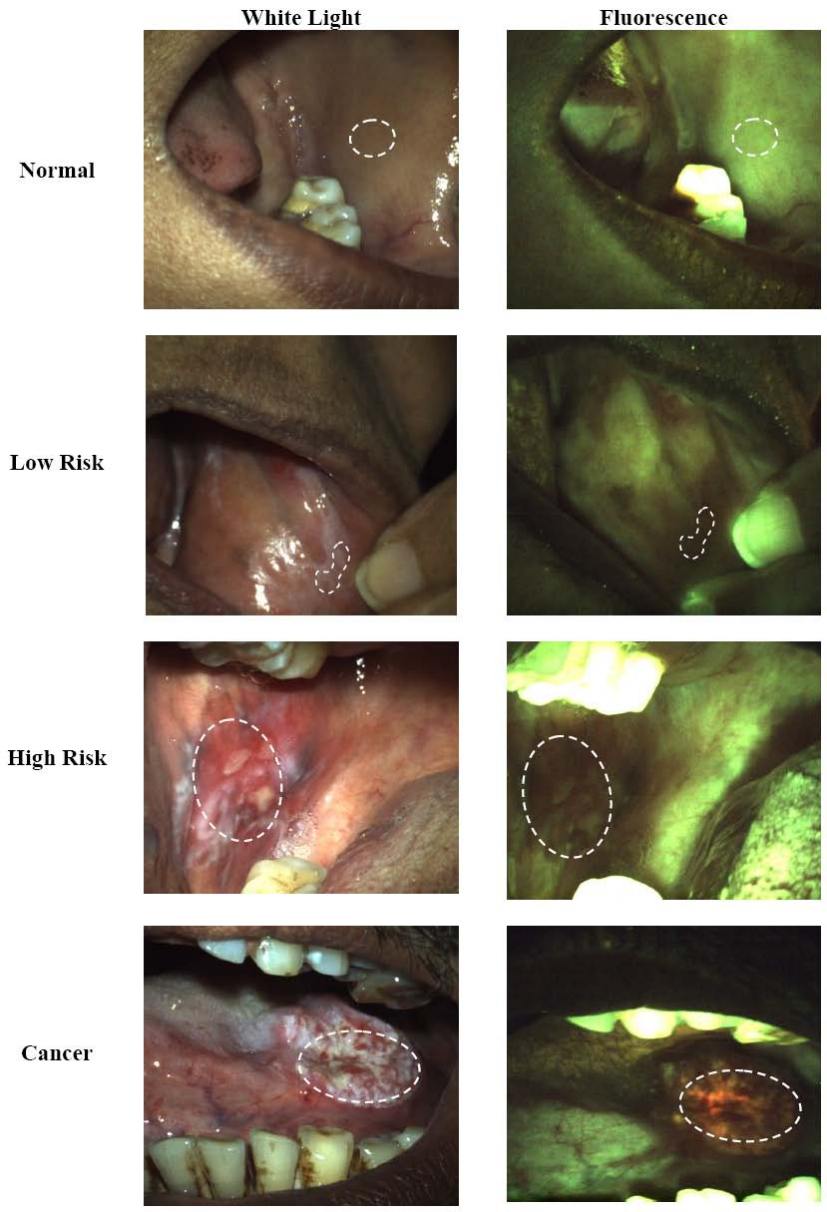
**Images of lesions typical of the four consensus clinical impression categories**. White light images are shown on the left and fluorescence images on the right. The top row illustrates 'Normal' buccal mucosa, illustrating homogenous green fluorescence. The second row shows a site with a 'Low Risk' lesion of the buccal mucosa, with loss of fluorescence. The third row shows a site with a 'High Risk' lesion in the buccal mucosa with loss of fluorescence. Bottom row illustrates 'Cancer' of the tongue, illustrating loss of green fluorescence and presence of orange-red fluorescence. Lesions are outlined in the image.

Figure 1 shows typical white light reflectance and fluorescence images of sites corresponding to each of the four consensus clinical impression categories. Fluorescence images of sites with a consensus clinical impression of 'Normal' showed homogenous green mucosal fluorescence. Sites with a consensus clinical impression of 'Low Risk' or 'High Risk' exhibited a progressive loss of green fluorescence, while sites with a consensus clinical impression of 'Cancer' typically showed both a loss of green fluorescence and an increase in orange-red fluorescence.

**Figure 2:**
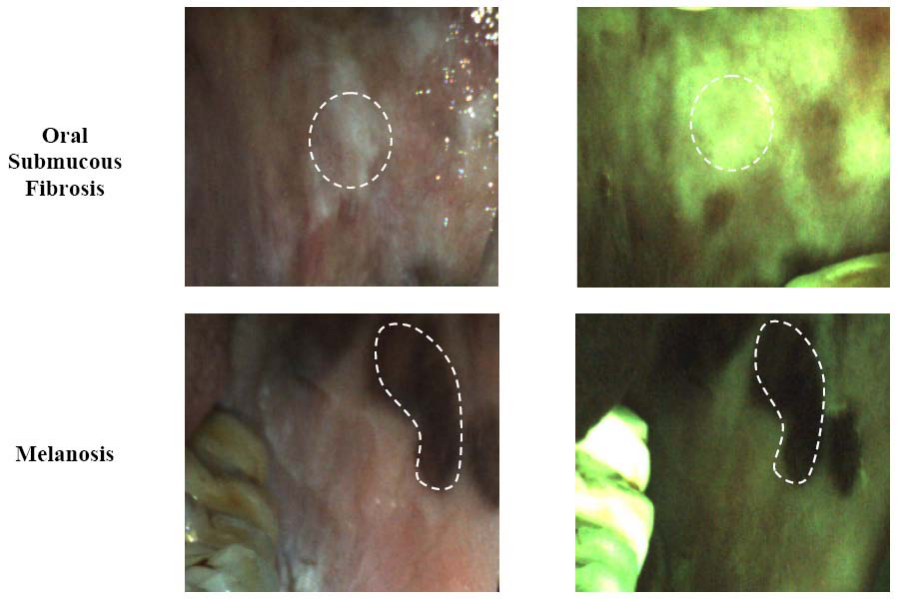
**White light images (left column) and fluorescence images (right column) of typical lesions with OSF (top row) and melanosis (bottom row)**. Areas with OSF show slightly increased green fluorescence. Melanosis is easily recognizable from the dark pigmentation in the white light image, and is associated with loss of fluorescence.

Figure 2 shows white light reflectance and fluorescence images of two potentially confounding lesions found in the study population - oral submucous fibrosis (OSF) and melanosis. In white light reflectance images, OSF sites exhibited a pale white, patchy appearance similar to leukoplakia. However, unlike leukoplakia which is often associated with decreased green fluorescence, no loss of fluorescence was observed in sites with a clinical description of OSF. Melanosis sites were easily recognized from the white light images as areas of dark pigmentation and were consistently associated with decreased green fluorescence.

Algorithms based on individual image features were ranked according to the area under the ROC curve (AUC); Table 2 lists the AUC for the five algorithms with the best performance relative to consensus clinical impression; all were based on normalized features of fluorescence images. The top three performing algorithms were based on the normalized mean fluorescence intensity (MFI) of the blue channel, the normalized MFI of the green channel, and the normalized ratio of red to green MFI. Linear discriminant analysis involving combinations of image features did not significantly improve classification performance.

**Table 2:** Area Under the ROC Curve (AUC) of Top Five Image Features for Binary Classification of Oral Sites

Features	AUC
Normalized MFI Green channel	0.91
Normalized MFI Blue channel	0.91
Normalized MFI Red/Green channel ratio	0.90
Normalized MFI Grayscale	0.89
Normalized MFI Red channel	0.85

MFI: Mean Fluorescence Intensity

**Figure 3:**
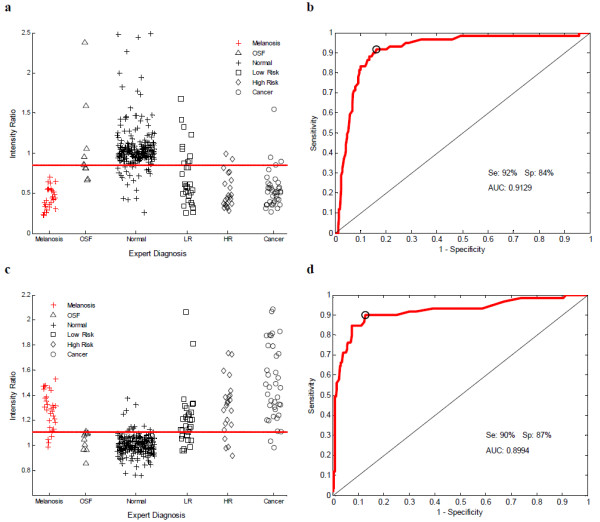
**Scatter plots of the image features and corresponding ROC curves for classification algorithms**. (a) Scatter plot of the normalized blue MFI for all sites measured by consensus clinical impression. (b) ROC curve for diagnostic algorithm based on normalized blue MFI to differentiate between non-neoplastic sites (Normal, LR and OSF) and neoplastic sites (HR and Cancer). Measurements with melanosis were excluded from algorithm development and testing. The open circle on the ROC curve indicates the Q-point. (c) Scatter plot of the normalized ratio of red to green MFI for all sites measured by consensus clinical impression. (d) ROC curve for diagnostic algorithm based on normalized ratio of red to green MFI to differentiate between non-neoplastic sites (Normal, LR and OSF) and neoplastic sites (HR and Cancer). Measurements with melanosis were excluded from algorithm development and testing. The open circle on the ROC curve indicates the Q-point.

Figure 3 shows scatter plots of the image features and corresponding ROC curves for classification algorithms which resulted in the best sensitivity and specificity, respectively. Figure 3a shows the normalized MFI from the blue channel by diagnostic category for all sites. Those sites with a consensus clinical impression of 'Normal' and clinically visible melanosis or OSF are shown separately. Sites with a consensus clinical impression of 'Low Risk', 'High Risk' or 'Cancer' exhibit decreased normalized blue MFI compared to 'Normal' sites. Sites with melanosis show decreased normalized blue MFI, while those with OSF have similar normalized blue MFI to other 'Normal' sites. Figure 3b shows the ROC curve for the algorithm based on normalized blue MFI, excluding melanosis from the analysis. The operating point indicated on the ROC curve corresponds to a sensitivity of 92% and a specificity of 84%; the threshold values corresponding to this operating point is a normalized blue MFI of 0.86, which is indicated by the solid horizontal line in Figure 3a.

Figure 3c shows a scatter plot of the normalized ratio of red to green MFI by consensus clinical impression for all sites. Sites with a consensus clinical impression of 'Low Risk', 'High Risk' or 'Cancer' exhibit an increased normalized red to green MFI ratio compared to 'Normal' sites; on average, the normalized red to green MFI ratio increases as the severity of the consensus clinical impression increases from 'Low Risk' to 'Cancer'. Sites with melanosis show an increased normalized red to green MFI ratio, while those with OSF have a similar normalized red to green MFI ratio as other 'Normal' sites. Figure 3d shows the ROC curve for the classification algorithm based on the normalized red to green MFI ratio, excluding melanosis from the analysis. The operating point indicated on the ROC curve corresponds to a sensitivity of 90% and a specificity of 87% relative to the gold standard of consensus clinical impression. The threshold value corresponding to this operating point is a normalized red to green MFI ratio of 1.11, and is indicated by the solid horizontal line shown in Figure 3c.

## Discussion

This pilot study demonstrates that objective analysis of fluorescence images obtained with a low-cost imaging system can classify sites as neoplastic or non-neoplastic with high sensitivity and specificity relative to the gold standard of consensus clinical impression. The performance of this low-cost, objective system compares favorably to results reported for pilot studies of other optical imaging systems. A recent review by De Veld summarizes several clinical studies of optical imaging with qualitative image analysis for detection of oral neoplasia and reports sensitivity ranging from 63% to 100% and specificity from 79% to 96% [14]. More recently, Lane et al. achieved a sensitivity of 98% and specificity of 100% using qualitative assessment of fluorescence images acquired with the VELScope to discriminate dysplasia and cancer from normal oral mucosa [5]. Roblyer reported quantitative analysis of fluorescence and reflectance images obtained with a multi-spectral digital microscope to classify oral premalignant lesions and oral cancer with a sensitivity of 94% and specificity of 87% [12]. Our results, obtained in a South Asian population using the low-cost, portable optical imaging system described here, demonstrate similar sensitivity and specificity.

It should be noted that most studies of optical imaging based adjunctive techniques for oral cancer detection have been carried out in high prevalence populations, and recent reviews stress the need for randomized controlled studies in low prevalence populations [15,16]. To be useful in low resource settings where the vast majority of oral cancers occur, screening aids must be affordable and must not rely on significant clinical expertise for image interpretation. Our results demonstrate the feasibility of conducting these much needed trials using low-cost, portable imaging devices in low resource settings.

The optical characteristics of oral lesions found in this South Asian population are similar to those in other studies. Our finding of decreased fluorescence associated with neoplastic lesions is consistent with reports in the literature. This loss of autofluorescence has been attributed to a decrease in collagen cross-links associated with neoplastic transformation [17]. Our results also indicate a relative increase in red fluorescence for neoplastic sites. Other studies have made similar observations, attributing this increased red fluorescence to porphyrins [8,9,17].

**Figure 4:**
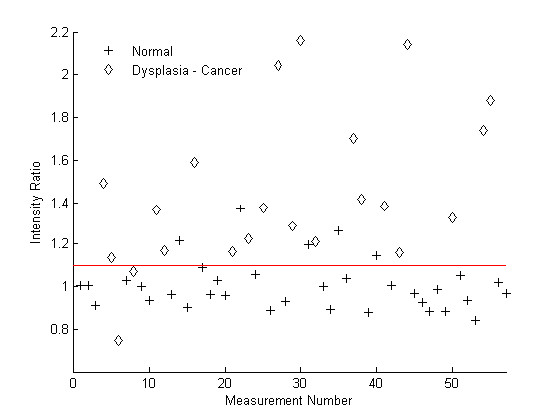
**Threshold ratio of multi-spectral digital microscope developed by Roblyer **[12]. Scatter plot of the normalized ratio of red to green MFI at 450 nm excitation with biopsy as gold standard using the multispectral digital microscope. The threshold value indicated by the horizontal line for differentiating premalignant lesions and cancer from normal sites is 1.09.

In addition, we found the normalized red to green MFI ratio to be amongst the top three best-performing features. Roblyer also reported that this parameter was the best performing feature for quantitative identification of oral premalignant lesions and oral cancer using a multispectral digital microscope [12]. Figure 4 presents the normalized red to green MFI ratio for 57 sites in 21 patients measured by Roblyer at 450-nm excitation; results are similar to those measured in this study (Figure 3c). Although the two studies were conducted independently on two entirely different populations and using two different instruments, the threshold ratio values from the two different systems, nevertheless, were very similar. The threshold to differentiate neoplastic sites from non-neoplastic sites is in good agreement between the two studies; here we found a threshold of 1.11 was optimal to differentiate neoplastic sites from non-neoplastic sites and Roblyer found a threshold of 1.09 could differentiate premalignant lesions and cancers from normal tissue.

The ability of fluorescence-based screening aids to differentiate precancerous lesions from benign lesions such as inflammation must also be validated in larger trials and the optical properties of other potentially confounding lesions, which may be population specific, must be characterized. For example, betel quid use is common in south Asia and is associated with a high incidence of OPLs as well as other potentially confounding oral lesions, including melanosis and oral submucous fibrosis (OSF) [18,19]. A recent study of 130 patients in the United States noted that 72% of lesions clinically characterized by inflammation or pigmentation show loss of fluoresecence with VELscope examination [20].

We characterized the optical properties of two types of potentially confounding lesions, melanosis and OSF, specific to the geographic region. Melanosis is usually benign and not considered to be precancerous [19]. Our results indicate that sites with melanosis exhibit decreased fluorescence, but can easily be recognized by their characteristic appearance in white light reflectance images. The loss of autofluorescence in melanosis is likely due to strong absorption of light by black pigmentation in the superficial epithelium. In contrast to melanosis, the malignant transformation rate of OSF has been estimated to be between 3% and 19% [21]. Most of the OSF sites measured in this study were graded as 'Low Risk'. We found that the OSF sites imaged here did not exhibit loss of fluorescence. Histologically, OSF is characterized by juxtaepithelial fibrosis, along with atrophy or hyperplasia of the overlying epithelium, keratinizing metaplasia, and accumulation of hyalinized collagen beneath the basement membrane [22-24]. All of these constituents are strong sources of autofluorescence and may contribute to autofluorescence observed in our images of OSF sites. The role of combined reflectance and fluorescence imaging with quantitative image analysis for better discrimination of inflammation and neoplasia should be further explored.

While the sensitivity and specificity reported here are encouraging, there are a number of limitations of this study. First, the same dataset was used both to develop classification algorithms and to assess their performance; in this situation, potential over-training can inflate estimates of sensitivity and specificity. Results must be verified in an independent validation set. Second, the gold standard used to assess algorithm performance was consensus clinical impression; due to resource limitations, histopathologic diagnosis was not available from all sites. Finally, a large number of 'Low Risk' sites were misclassified as neoplastic by the optical algorithms presented here. It is interesting to note that 'Low Risk' sites with consensus among all three expert observers were more likely to be classified by the optical algorithms as non-neoplastic (6/10 = 60%) than were 'Low Risk' sites where only two of the expert observers agreed (13/30 = 43%). Additional studies with histologic endpoints for all sites are required to assess the ability of quantitative optical image analysis to aid in the evaluation of low risk oral lesions.

## Conclusions

The clinical study presented here demonstrates the ability to identify neoplastic and non-neoplastic oral tissue *in vivo *objectively using the low-cost, portable imaging system. Although further work is needed to address the limitations in this study, results from this pilot study suggest that this simple imaging device can potentially improve oral screening efforts in low-resource settings where clinical expertise and resources are often limited.

## Competing interests

Dr. Richards-Kortum serves as an unpaid scientific advisor to Remicalm LLC, holds patents related to optical diagnostic technologies that have been licensed to Remicalm LLC, and holds minority ownership in Remicalm LLC. Dr. Gillenwater holds patents related to optical diagnostic technologies that have been licensed to Remicalm LLC, and serves as an unpaid scientific advisor to Remicalm LLC.

## Authors' contributions

MSR participated in the design of the study, carried out the clinical measurements, analyzed data and drafted the manuscript. NI recruited the subjects and provided clinical background for the measurements. DR provided analysis tool to develop classification algorithm. VS provided clinical background for the measurements. RR contributed to conception and design of the study, and provided critical review of the manuscript for important intellectual content. AG conceived of the study, participated in its design and provided clinical background for the measurements. SS participated in coordination and design of the study. PC participated in the design of the study and coordination, and provided clinical background for the measurements. All authors read and approved the final manuscript.
